# Predicting construction waste in Egyptian residential projects: a robust multiple regression model approach

**DOI:** 10.1038/s41598-025-86474-1

**Published:** 2025-01-23

**Authors:** Mohamed KhairEldin, Ahmed Osama Daoud, Ahmed Hussein Ibrahim, Hossam M. Toma

**Affiliations:** 1 Construction Engineering and Utilities Department, Faculty of Engineering, Zagazig University, Zagazig, 44519 Egypt; 2 Civil Engineering Department, Faculty of Engineering, The British University in Egypt (BUE), El Sherouk City, Cairo, 11837 Egypt

**Keywords:** Construction waste, Waste prediction, Concrete, Bricks, Steel, Civil engineering, Statistics, Sustainability

## Abstract

Effective construction waste (CW) management, mainly concrete, brick, and steel, is a critical challenge due to its significant environmental and economic impacts. This study addresses this challenge by proposing multiple linear regression models to predict waste generation in residential buildings within the Egyptian construction industry, considering the influence of factors such as building design and site management features. Using data from 25 case studies, the models demonstrated high predictive accuracy, with adjusted R² values of 0.877, 0.893, and 0.889 for concrete, bricks, and steel waste, respectively. These R^2^ values indicate that the models explain approximately 88–89% of the variance in waste generation in residential buildings, highlighting their effectiveness in enhancing resource planning and waste management strategies. The findings suggest that incorporating variables such as total area, design consistency, and site organization significantly improves the accuracy of waste predictions. Although the models show acceptable performance, future research should aim to expand the dataset, incorporate additional variables, and test the models across different types of construction projects to validate further and refine these predictive tools. The models offer valuable insights for enhancing construction practices, minimizing waste, and supporting sustainable development in Egypt’s construction industry. With accurate forecasts of waste generation, the models help project managers and stakeholders to plan CW more effectively, mitigating unnecessary material consumption and reducing environmental impacts. These findings help to adopt sustainable construction practices, such as improved recycling processes and decreased dependence on landfills, to support Egypt’s Vision 2030.

## Introduction

The construction sector is forced to adhere to sustainability standards because of its significant consumption of raw materials and considerable generation of waste^1^. In developing countries, balancing environmental conservation and economic advancement is a common challenge due to environmental and economic weakness^2^. For instance, Egypt’s construction sector, as one of the key providers of the economy’s growth sources, is a major contributor to gross domestic product but also generates about 35–40 million tons of construction and demolition waste, increasing by 5 million tons each year^3^. This challenge increases in the construction industry, creating essential infrastructure to support economic growth while producing significant waste^4^. Construction waste (CW) represents a significant portion of all solid waste generated worldwide, accounting for approximately 35%^5,6^. Tafesse et al.^7^ reported that nearly 95.71% of ongoing construction projects face substantial challenges due to CW. Globally, the generation of CW leads to excessive consumption of raw materials, environmental pollution, cost overruns, reduced profits, bankrupt construction firms, and risks to public health and safety^7,8^. CW accumulation has destructive effects on the economy, environment, and society. They consume landfill space, pose geological and ecological risks, accelerate land depletion, and lead to negative impacts^9,10^.

The waste management guidelines emphasize eliminating waste generation by adopting processes that minimize or avoid waste generation altogether^11–13^. A secondary strategy involves reusing, recycling, recovering, and ensuring proper waste disposal^14–16^. Despite the potential to minimize CW using these strategies, there is a prevailing tendency among practitioners and workers to dispose of waste in landfills^17^. However, if this trend continues, landfills may soon be insufficient to fulfill the increasing demand. Despite the negative consequences of poor waste management practices, the construction industry has not given sufficient attention to managing CW effectively^18^. According to Maués et al.^6^, CW seriously damages the natural environment and quality of life without proper management. Recent literature highlights the importance of reducing CW throughout the different stages of a project, ranging from design and material procurement to the construction phase^19^. While several waste reduction strategies have been proposed for every stage of the building process, previous studies have revealed that measures to CW should be applied early in the project lifecycle to manage CW effectively^20,21^. One of the main approaches to waste management is the quantification and prediction of CW, as these processes enable industry stakeholders to take appropriate actions at the right time, minimizing and managing CW generation effectively^18^.

Despite several waste reduction and management strategies adopted worldwide, their implementation remains inconsistent, especially in regions with less developed infrastructure. In Egypt, for instance, CW is often viewed as an unavoidable byproduct of building activities rather than an opportunity for resource recovery and reuse^22–24^. This perspective restricts adopting more sustainable practices and limits the effectiveness of existing waste management frameworks. Moreover, the lack of reliable waste generation data in the Egyptian context has posed a significant barrier to developing accurate predictive models^25,26^. This study seeks to address the gap in predictive modeling for CW in Egypt, where localized data-driven models are rare, by using multiple linear regression models to predict waste generation, focusing on crucial construction materials such as concrete, brick, and steel. Table 1 shows the main challenges in developing prediction models of CW in Egypt and their impacts.

**Table 1:** Main challenges in developing CW prediction models.

Challenges	Impact
Lack of CW generation data	Reduces the precision and effectiveness of predictive models
Numerous factors associated with construction processes	The properties of CW generation cannot be accurately identified when using a small number of input variables. Also, using many input variables may cause overfitting issues
Low awareness of waste management principles	Data inconsistency may hinder the model’s accuracy

This study focused on concrete, brick, and steel as critical materials for studying CW in Egypt. These materials were chosen due to their widespread use in construction projects, high potential for recycling and reuse, relevance to regulatory frameworks, and their significant impact on the economic and environmental impression of the construction industry. The study aims to provide insights into waste management practices that can contribute to more sustainable construction practices in Egypt by analyzing these materials. According to El-Desouky et al.^27^, bricks are the most wasted material in Egypt. Daoud et al.^22^ also mentioned that concrete, brick, and steel are among the components that contribute to waste in residential buildings in Egypt.

The objective of this research is to predict the amount of concrete, brick, and steel waste generated during the construction of residential buildings, taking into account the influencing factors related to the design of the building and site management attributes. A quantitative approach utilizing multiple regression analysis is employed in this study to predict the amount of CW, as done by Parisi Kern et al.^28^. Due to the large number of influencing factors and the complexity involved in adjusting regression models, predictions in this context require further investigation, especially when considering different types of CW. The factors that influence CW generation vary depending on the types of waste being considered. Therefore, the dependent variables studied in this study are specific and include analyses of building design and site management characteristics, and each type of waste is investigated individually.

## Literature review

### Construction waste quantification methods

Waste quantification measures and counts the total generated waste from a specific project at different stages and is expressed in statistical datasets. One of the performance indicators for the waste management implementation plan is waste quantification, which can also be used to monitor the overall success of a project^29^. Researchers such as Lu et al.^30^ and Li et al.^31^ have observed that the unit of analysis for estimating CW generation could be a project or a region. For instance, Lu et al. (2017) examined the CW generation at the regional level by dividing China into zones with similar characteristics. Although the strength of this approach is that it gives a general idea of the waste trends and effects of regional policy, their study did not consider the specific factors of each project. In this research, the unit of analysis is a project primarily because of its well-defined system boundaries. Results from these projects can then be aggregated to estimate the waste generation on broader scales, such as regional levels. This approach enables a comprehensive understanding and estimation of CW across larger geographic areas.

Two methods are utilized to quantify the site’s waste generation amounts or rates^32^. There are hard measures and soft measures. The hard measure methods include the Material Flow Analysis Approach (MFA) and Sorted and Weighed Waste Materials. The soft measure method is Interviews and Questionnaires and Estimation Based on Statistical Data^33^. Wu et al.^34^ further classified waste estimation methods into six categories based on waste generation activity, estimation level, and quantification methodology, including site visits, waste generation rate, lifetime analysis, classification accumulation, variables modeling, and other specific methods. These methods could be used separately or combined in the real-life estimate^35^.

### Construction waste prediction models

Several studies have been conducted to predict the amount of CW using regression analysis and artificial intelligence techniques. Sáez et al.^36^ analyzed seven building projects, evaluating waste volume and weight at construction sites. According to the coefficients of determination (R2) values in both models equivalent to 0.99, only a tiny percentage of the variation in waste (1%) cannot be accounted for by combining the two variables: the number of dwellings and the total floor area. The model-derived estimates showed a 10% mean deviation from the measured values. A correction factor depending on the built-up area and number of dwelling units was included in the model. This factor appeared within the model’s linear, quadratic, and cubic forms. Using a small sample may pose several challenges regarding the validity and applicability of the data obtained and their generality. The analysis was also made using two variables to avoid overfitting. Parisi Kern et al.^11^ studied eighteen high-rise building projects and proposed a statistical model by assessing the influence of the design process and production system. The proposed linear model demonstrated satisfactory statistical performance (R^2^ = 0.694), with all attributes approved on the t-tests at the 5% significance level. The model showed the relationship between waste and several variables, such as design variables: floor area, the ratio between the number of apartments and floors, the wall density, and the economic compactness index. The collected variables associated with production were the construction system, site organization, and waste reuse.

Teixeira et al.^37^ developed two regression models to forecast the amount of CW based on analyzing 18 building sites. The first model was designed to account for total waste generation, incorporating specific attributes, achieving an R^2^ of 0.81. It estimated changes in waste produced during construction. The second model incorporated time schedules and analyzed how the construction stage influenced waste generation, achieving an R^2^ of 0.91. This model demonstrated an S-shaped relationship with time. Some of the factors used in this study were total built area, number of floors, reuse or recycling of waste onsite, and site layout.

Hassan et al.^38^ developed a linear regression model for estimating the quantity of brick waste generated in Malaysia. Considering the correlation between the area of work and the amount of brick waste, the regression model developed from the sample data yielded an R-squared value of 0.78. This study indicated that the primary generators of brick waste were typically unskilled workers assigned to skilled work.

In addition to regression, various other methods have been utilized to develop models. Maués et al.^6^ developed a fuzzy set theory model to predict the amount of CW generated in the Brazilian Amazon. This model was calibrated and validated based on a case study involving 23 residential buildings. The model followed three methodology steps: model development, sensitivity analysis, and validation. Based on the built area and number of floors as independent variables, a set of IF–THEN rules was developed, and the model’s classification of waste generation level was accurate at a rate of 66.67%. A limitation of this model is its generalizability. It cannot be applied to other localities or construction projects since the volume of waste residuals varies greatly depending on each region’s cultural characteristics and construction techniques.

Gulghane et al.^29^ developed a machine-learning model to predict the amount of CW generated at different stages of construction projects. The generated waste data from 134 construction sites was collected. The decision tree and the K-nearest neighbor’s method were utilized for analysis, and the performance of neural networks was assessed by providing total floor area and material estimation. The model had an average RSME value of 0.49, indicating that its accuracy was acceptable for making predictions. The combined average accuracy of the decision tree and KNN was 88.32% and 88.51%, respectively.

Elshaboury and AlMetwaly^39^ developed a comprehensive methodology to forecast CW generation in Tanta City, Egypt, utilizing socio-economic and waste generation data from 1965 to 2021. Hybrid fuzzy neural network techniques were used. Their study included factors such as the year, population, gross domestic product per capita, built-up area, vacant area, and agriculture area. However, this approach did not incorporate several significant factors related to CW generation.

Maged et al.^2^ developed a reliable method to predict CW generation with limited data. Optimization algorithms were applied to extreme gradient boosting and compared against standard machine learning models, such as artificial neural networks (ANN), support vector regression (SVR), and decision trees. The developed model achieved outstanding results, demonstrating testing R^2^ values as high as 99.9%. Table 2 shows the studies reviewed in this paper.

**Table 2:** Previous studies on waste generation models.

Model	Strengths	Weaknesses
Sáez et al.^36^	The research methods offered an analytical framework, even with limited data, to assess the building-related CW generation in China	The model used a small sample size and a small number of variables
Parisi Kern et al.^11^	Proposing a statistical model by assessing the influence of the design process and production system	The quantity of CW used in the models didn’t depend on actual measures but was based on theoretical assumptions
Teixeira et al.^37^	High predictive accuracy with R^2^ values of 0.81 and 0.91	The measurements taken on site were of mixed waste. As a result, it could be challenging to differentiate between the kind of waste most prevalent in each stage of the construction process
Hassan et al.^38^	The model can predict roughly 78% of the factors concerned in brick waste generation	Stated that the primary generators of brick waste were typically unskilled workers and didn’t take their effect when developing the model
Maués et al.^6^	Developing a model that can be utilized to estimate CW generation based on fuzzy set theory	A limitation of this model is the small number of variables and its generalizability. It cannot be applied to other construction projects
Gulghane et al.^29^	Developing a machine-learning model to predict the amount of CW generated at different stages of construction projects	The factors used in this study were only total floor area and material estimation
Elshaboury and AlMetwaly^39^	Developing a comprehensive methodology to forecast CW generation in Tanta City, Egypt	The model was applied to Tanta city only and may require justification when applied to other cities with varying characteristics
Maged et al.^2^	These research results can be used to strategically plan waste treatment infrastructure, observe the metabolism of urban spaces, and build a circular economy system with scarce data	It is inaccurate to forecast future waste generation using the currently available data

Currently, there are several issues with the data collection infrastructure in Egypt, particularly in the construction and waste management industries. These deficiencies restrict the acquisition of dependable, consecutive, and integrated data, which is fundamental for prediction models of CW generation. The lack of historical data on CW generation makes it challenging to develop prediction models. A framework or standardized methodology does not define CW generation data collection. As a result, data is measured, recorded, and reported differently on different projects in different regions. Sensors, automated tracking systems, and Geographic Information Systems (GIS) are underutilized, reducing the accuracy and efficiency of data collection. Also, the low awareness of the importance of data collection for waste prediction and management among stakeholders makes it difficult to predict CW generation. In reviewing the current literature on CW prediction, it becomes evident that there is a significant gap in predictive modeling for the Egyptian construction industry. Also, the lack of historical data on CW quantities and targeted research on CW prediction models in Egypt restricts the ability to predict and manage waste accurately. This lack of localized models and data restricts the development of effective waste management strategies and policies suited to the specific conditions of the Egyptian construction sector.

## Research method

**Fig. 1:**
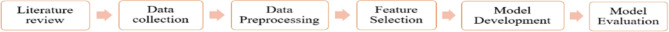
The research study’s flowchart.

The flowchart presented in Fig. 1 illustrates the detailed steps involved in developing the linear prediction model. Multiple linear regression analysis was utilized as a research method to achieve the stated research objective of developing a model to forecast CW quantities in residential buildings. Multiple linear regression is frequently used to determine the relationship between predictor variables and waste generation^11,40–42^. A case study was used in the research, which included collecting quantitative and qualitative data on waste generated from various construction companies during the construction of buildings.

The study employs a quantitative research design, utilizing multiple linear regression analysis to predict the generation of construction waste in residential buildings. This approach was selected due to its effectiveness in modeling relationships between a dependent variable and multiple independent variables, which is particularly useful in construction waste estimation^31^. Multiple linear regression is widely regarded as a robust statistical method for quantifying the impact of various design and site management factors on waste generation, allowing for identifying key determinants contributing to higher levels of waste^11,28^.

### Characteristics of the sample

**Fig. 2:**
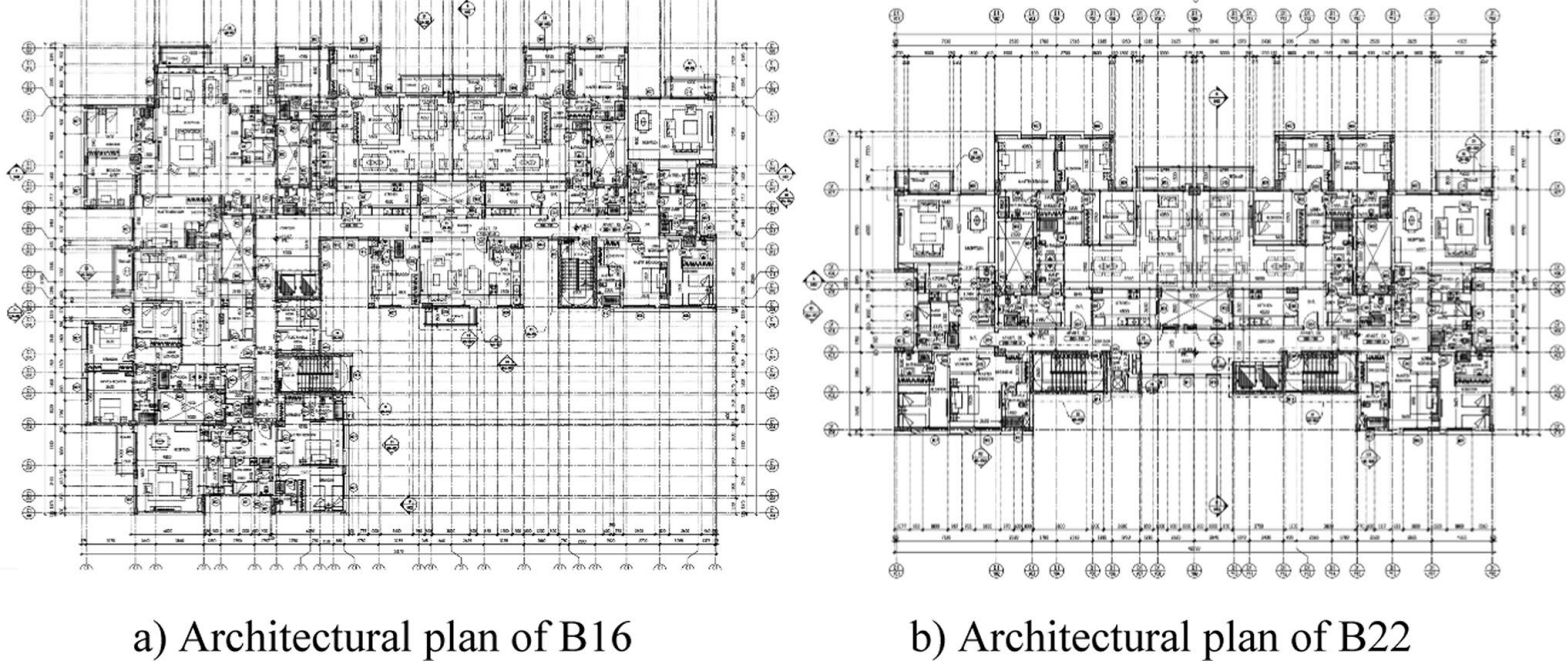
Architectural floor plan for (**a**) B16 and (**b**) B22.

This study focused only on residential buildings, as Egypt is witnessing rapid urban expansion, including the development of vacant areas and the construction of new urban communities, such as city capital development projects^43^. Data collection for this study involved gathering information from 25 residential buildings across various urban areas in Egypt regarding the amount of CW generated. The sample number is accepted according to similar studies, such as those by Parisi Kern et al.^11^, Hassan et al.^38^, and Teixeira et al.^37^ that have used small sample sizes, and it is still possible to obtain relevant information from the results, which implies the validity of this approach. As shown in Fig. 2, the selection of projects was based on the variety of building architectural designs and construction practices, ensuring that the sample represents the broader construction industry in Egypt. Data collection involved site visits, interviews with project managers, and the analysis of construction records to obtain accurate measurements of waste generated. This multi-method approach is essential for capturing the complexity of construction processes and improving the reliability of the data used in the regression analysis^44^.

The buildings were labeled B1 to B25. To explore the impact of design on waste generation, buildings with varied floor plans were explicitly chosen for analysis. All the buildings in the sample are residential structures; the number of floors ranges between 3 (B21) and 13 (B1). The total area of the building ranges from approximately 1140 to 16,456 m^2^. The structural systems comprised reinforced concrete structures (columns, beams, and slabs) and masonry walls. As CW management practices are still in the early stages of development in Egypt, particularly concerning waste reduction, information gathered from architects and constructors indicates that the studied construction companies have not implemented formal design or construction briefs that include specific requirements or targets for waste reduction. Unfortunately, waste is still perceived more as an undesirable outcome of construction activities than a potential opportunity for beneficial outcomes.

### Dependent variable

It is difficult to estimate the amount of CW generated accurately, especially in Egypt, where many buildings are unplanned and in slum areas. It is also common for local contractors and developers to lack proper systems for managing CW or to register waste at the site. As a result, there is often no reliable record of the waste generated during construction activities. The amount of CW generated by each project was recorded based on the loading capacity of waste-hauling trucks used for transporting CW, interviews with project managers, and observations conducted at the construction sites. The total quantity of concrete, brick, and steel waste collected from each selected building was utilized as the dependent variable and measured in weight (ton).

### Independent variables

This research determines the variables that predict CW generation based on the literature and data availability. These variables encompass design-related factors, such as total floor area and wall density, and site management attributes, including site organization and material storage practices. Previous studies have demonstrated that these factors significantly influence the amount of waste generated on construction sites, as demonstrated in the literature review (“Construction waste prediction models” section). Additionally, insights from the researchers have been incorporated to add more variables, such as design consistency and workers’ experience. By integrating these variables into the regression model, the study aims to develop a more accurate and context-specific prediction tool for the Egyptian construction industry.

The independent variables in this study included design process and site management attributes:

- Design factors such as total area, number of floors, wall density, and design consistency (reflecting both the absence of errors and the minimization of changes in the design) influence the waste amount together.
- Site management factors include site organization, practices related to waste reuse onsite, material storage, and workers’ experience.

#### Design variables

The quantitative variables, except for design consistency, are associated with architectural designs derived from the building’s design and specifications. Following is a description of design variables:

1. “Total area” refers to the building’s total constructed area as shown in the drawings (m^2^), and the value assigned for this variable is fixed for concrete, brick, and steel prediction models.
2. “Number of floors” refers to the building’s number of floors, and the value assigned for this variable is fixed for concrete, brick, and steel prediction models.
3. “Wall density” refers to the floor walls’ length divided by the area of the floor (m/m^2^), and this variable is used only for the brick prediction model.
4. “Design consistency” (yes/no): yes, if there are no changes or errors in the design, the value assigned for this variable is fixed for concrete, brick, and steel prediction models.

#### Site management variables

Although qualitative, variables related to the site management attributes need to be converted into numerical representations for statistical analysis. This conversion allows for quantitative assessment and comparison across different site management attributes, facilitating strict statistical analysis and interpretation of the data. Visits to the construction sites were conducted to gather data on waste generation and assess other relevant attributes during various phases of construction. This procedure was established to monitor site organization, the actual management strategies, and construction methods implemented by these firms onsite. The score was derived from the perceptions of the researchers and previous research, such as Karunasena et al.^45^ and Parisi Kern et al.^11^. The collected variables associated with site management attributes are:

- “Site organization” was evaluated on a scale of 1–5, considering factors including cleanliness, security, quality, and management tools. The score was assigned as (1) significant organization problems across all aspects; (2) organization problems observed in some aspects; (3) intermediate organization level, with problems in some aspects; (4) few organization problems noted; and (5) excellent organization observed in all aspects. The value assigned for this variable is fixed for concrete, brick, and steel prediction models.
- “The experience of workers” was assessed on a scale from 1 to 4. This scoring system allowed for the categorization of workers based on their level of experience and was based on subjective perceptions by researchers: (1) workers with less than four years of experience; (2) workers with four to ten years of experience; (3) workers with 11 to 15 years of experience; and (4) workers with more than 15 years of experience. The value assigned for this variable varies across concrete, brick, and steel prediction models.
- “Storage of materials” (yes/no): yes, if the construction materials are effectively stored onsite. This variable is not used for the concrete prediction model because pumps do not require onsite storage of poured concrete. The value assigned for this variable is fixed for brick and steel prediction models.
- “Waste reuse” (yes/no): if the construction waste is reused onsite in quantities equal to 10–15% of the overall waste. The value assigned for this variable varies across concrete, brick, and steel prediction models.

### Statistical data analysis

The regression analysis was conducted using IBM SPSS software, which is well-suited for handling complex datasets and performing multiple linear regression analysis. The collected data was processed using multiple regression to study the relationship between the dependent variable Y (the generation of waste) and the independent variable X (design and site management attributes). The dependent variable’s normality was tested using the Kolmogorov–Smirnov and Shapiro–Wilk tests, commonly applied in studies with small to medium sample sizes below 30^46–48^. These tests, along with scatter plots and boxplots, were used to assess the distribution of data and identify potential outliers, ensuring the validity of the regression model.

The variables that affected the generation waste (Y) were then subjected to regression analysis. The analysis included R^2^, adjusted R^2^, correlation (r), tests of the explanatory variables (t), and analysis of variance (F)^49^. The final model’s accuracy was evaluated using the adjusted R^2^ value, which indicates the proportion of variance in waste generation explained by the independent variables. The final model estimates the amount of concrete, brick, and steel waste in the 25 buildings compared with the amount collected from the construction sites.

## Results

The results are structured into four parts. The first part of the results is the assigned values for the variables in each sample. The concrete, brick, and steel prediction models follow this.

### The assigned values for the sample

The dependent variable (Y) is the amount of waste, which includes the quantity of each type of waste generated in the buildings, as listed in Table 3. The independent variables (X) are the design and site management attributes identified in Table 4, which include wall density (m/m^2^), design consistency (yes/no), site organization (rated on a scale from 1, indicating severe organizational problems, to 5, indicating optimal organization), and the efficiency of material storage (efficient/inefficient).

**Table 3:** The amount of waste generation in the buildings.

Sample	Waste (ton)		
	Concrete	Steel	Brick
B1	220	8	70
B2	82	2.1	27
B3	229	4	49
B4	42	0.8	17
B5	90	2.3	31
B6	319	8.1	98.5
B7	146	4.8	55
B8	141	3.8	46.5
B9	230	7.2	72
B10	39	1.1	14.4
B11	180	5.8	66.5
B12	196	7	78
B13	229	7.3	90.5
B14	460	17.1	106
B15	240	6.4	65
B16	251	15	115
B17	238	7.9	65
B18	420	15.7	120
B19	280	9.6	110
B20	40	1.5	26
B21	47	1.7	20
B22	162	4.7	60
B23	186	6	80
B24	137	2.5	40
B25	155	10.5	111.5

**Table 4:** Design and site management variables.

Sample	Total area (m^2^)	Floors (#)	“Wall density” (m/m^2^)	“Design consistency”	“Site organization” (1 to 5)	“Storage of materials”
B1	8151	13	0.57	No	2	Efficient
B2	2670	7	0.51	Yes	4	Inefficient
B3	3532.20	6	0.41	Yes	1	Efficient
B4	1140	4	0.54	Yes	4	Efficient
B5	2864.4	6	0.65	Yes	3	Efficient
B6	9810	9	0.66	Yes	4	Inefficient
B7	4770	10	0.55	No	3	Inefficient
B8	4246	6	0.47	Yes	1	Efficient
B9	8280	9	0.41	Yes	2	Inefficient
B10	1362	5	0.5	Yes	3	Inefficient
B11	5394	6	0.59	No	2	Efficient
B12	6358	7	0.6	No	3	Efficient
B13	5250	7	0.64	Yes	2	Efficient
B14	11,064	12	0.54	No	3	Inefficient
B15	7014	10	0.4	Yes	2	Inefficient
B16	8763	7	0.59	No	2	Inefficient
B17	6856	6	0.51	No	3	Efficient
B18	16,456	11	0.48	Yes	3	Inefficient
B19	9810	9	0.66	No	4	Inefficient
B20	2066	6	0.6	Yes	4	Efficient
B21	1520	3	0.6	Yes	4	Efficient
B22	4752	6	0.49	No	3	Efficient
B23	5616	8	0.57	No	1	Inefficient
B24	3285.7	5	0.61	Yes	2	Efficient
B25	9100	10	0.52	No	3	Efficient

It is important to note that Table 5 includes variables that differ across various prediction models, precisely the number of experienced workers (rated from 1 to 4) and the practice of waste reuse (categorized as yes or no). These variables play a significant role in influencing the outcomes of different models.

**Table 5:** Variables with varying values across prediction models.

Sample	Concrete waste		Steel waste		Brick waste	
	“Experienced workers” (1 to 4)	“Waste reuse”	“Experienced workers” (1 to 4)	“Waste reuse”	“Experienced workers” (1 to 4)	“Waste reuse”
B1	2	No	2	Yes	2	Yes
B2	3	Yes	3	Yes	4	Yes
B3	2	No	3	Yes	3	No
B4	4	Yes	4	Yes	4	Yes
B5	3	Yes	3	Yes	2	Yes
B6	1	No	2	No	3	No
B7	4	Yes	3	Yes	3	Yes
B8	3	Yes	3	Yes	3	No
B9	2	No	2	No	3	No
B10	4	Yes	2	Yes	4	No
B11	3	No	3	Yes	2	No
B12	3	No	2	Yes	2	Yes
B13	1	No	3	No	1	No
B14	1	Yes	2	Yes	2	No
B15	2	No	2	No	3	Yes
B16	2	No	1	No	2	No
B17	2	No	2	No	2	Yes
B18	3	Yes	2	No	4	Yes
B19	4	No	1	No	2	No
B20	2	Yes	4	Yes	3	Yes
B21	3	Yes	3	Yes	2	Yes
B22	3	No	3	Yes	3	Yes
B23	2	Yes	2	No	3	Yes
B24	1	No	3	Yes	2	Yes
B25	2	Yes	1	No	1	No

### Concrete prediction model

#### Statistical analysis

**Fig. 3:**
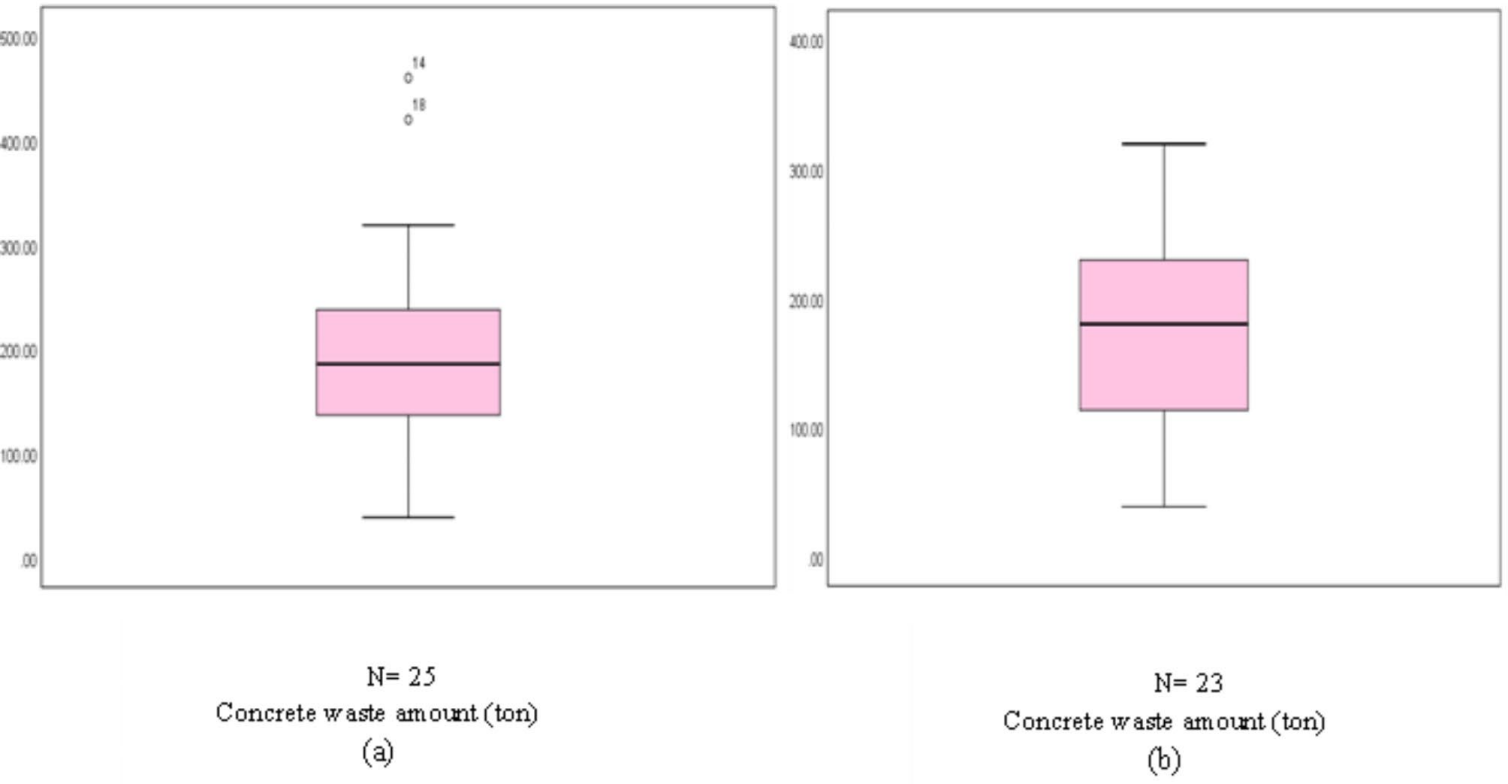
(**a**) Box plot of the 25 buildings, and (**b**) box plot of the 23 buildings.

Table 6 shows the normality test results, which included a sample of 25 buildings. The p-value of 0.123 indicates that the dependent variable’s normality hypothesis was not rejected. The existence of outliers was then examined. Hair et al.^50^ identified three types of outliers: events, process errors, and unusual observations. The box plot (Fig. 3a) revealed the presence of two outliers, B14 and B18, in the first sample (25 buildings). After identifying and removing outliers from the dataset, the normality tests of 23 building samples were conducted, with a p-value of 0.223. Therefore, the normality hypothesis of the dependent variable was not rejected. There are no outliers in the 23 building samples, as indicated by the box plot in Fig. 3b. Table 6 compares the two sample sets’ normality tests before and after removing outliers.

**Table 6:** Dependent variable’s normality tests (25 and 23 building samples) for concrete.

	Kolmogorov–Smirnov			Shapiro–Wilk		
	Statistic	df	Sig.	Statistic	df	Sig.
25 buildings						
Concrete waste amount	0.128	25	0.200	0.937	25	0.123
23 buildings						
Concrete waste amount	0.126	23	0.200	0.944	23	0.223

**Fig. 4:**
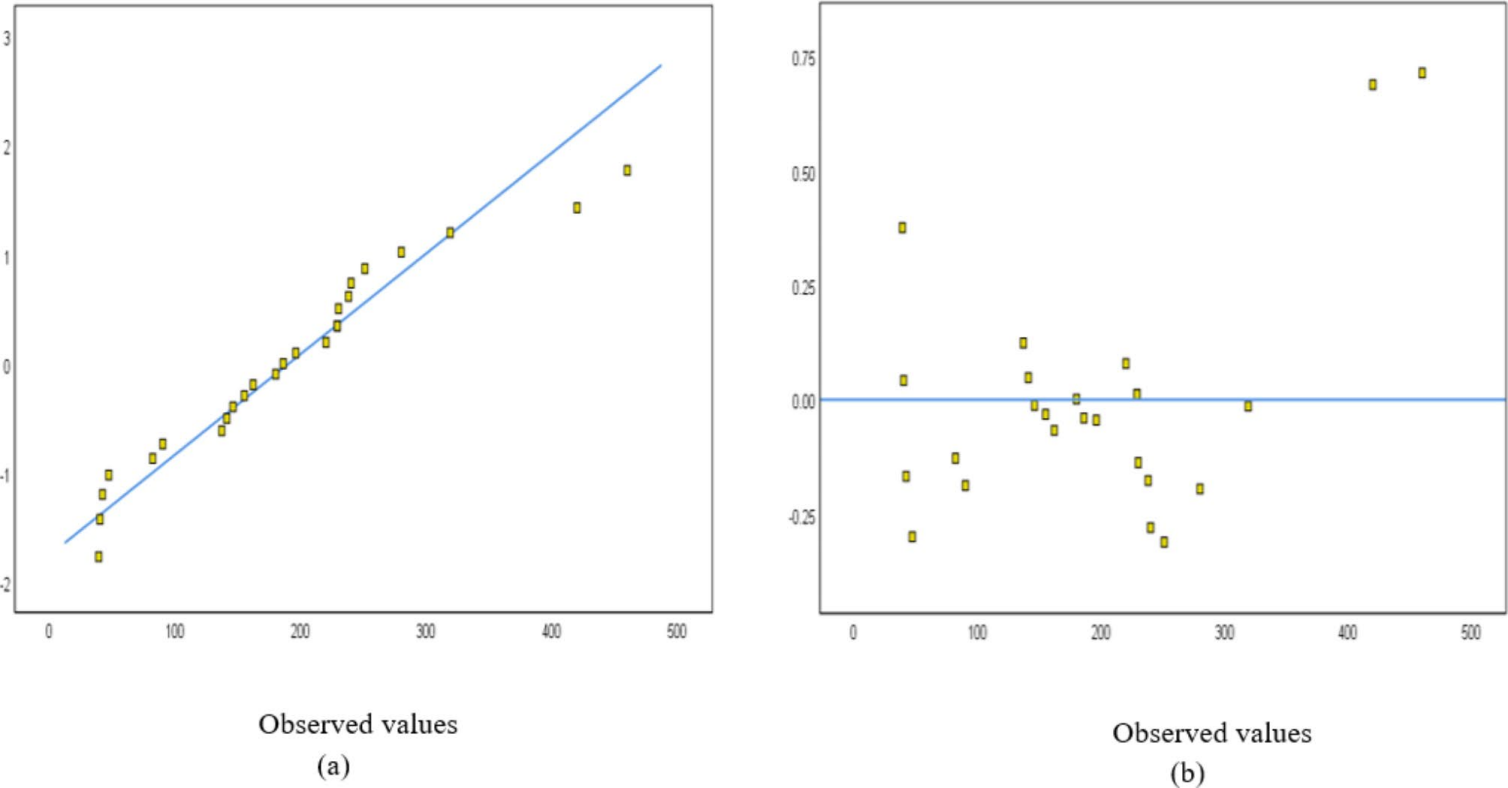
A sampling of 25 buildings: (**a**) Normal probability and (**b**) detrended Normal Q–Q Plot for concrete.

**Fig. 5:**
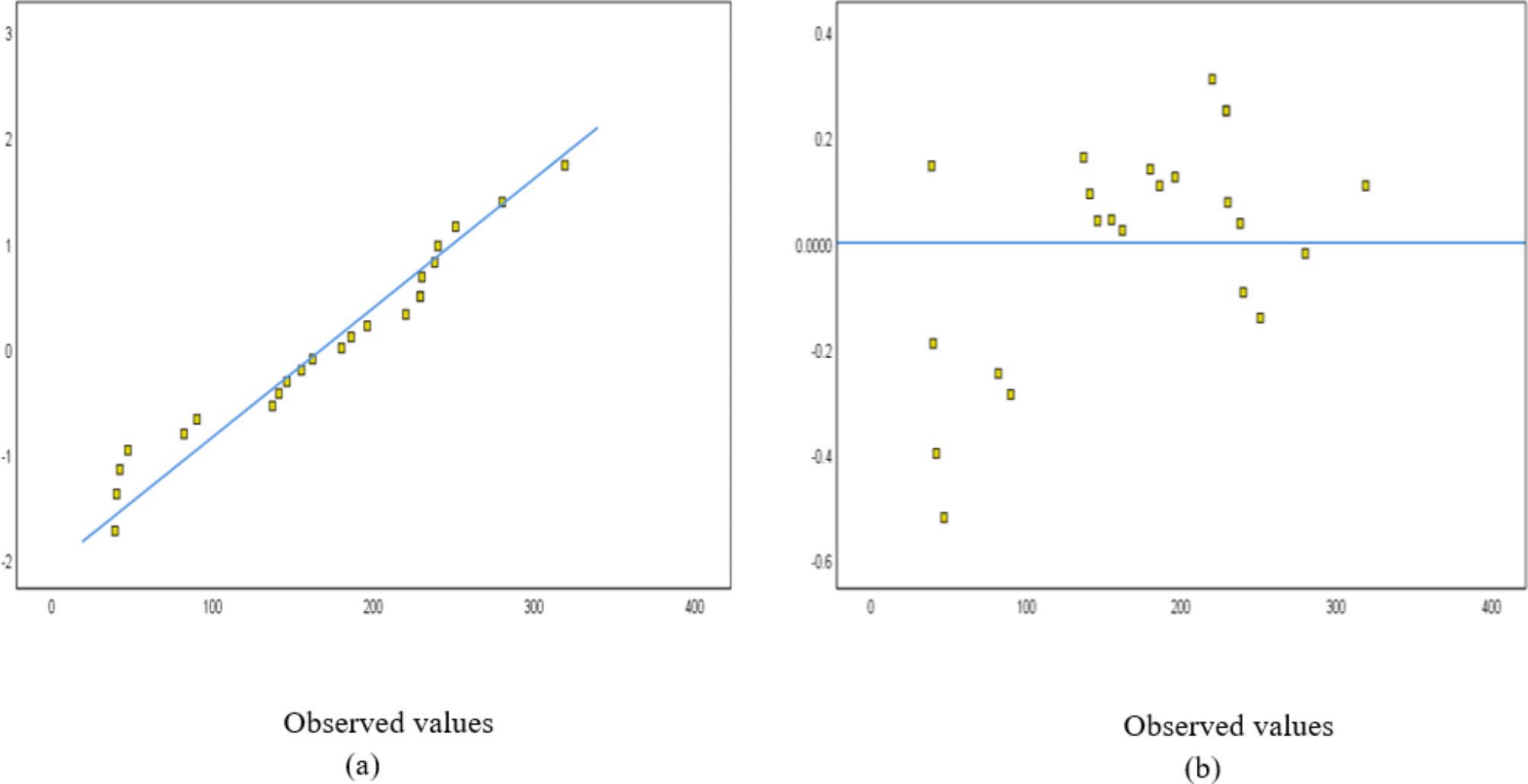
A sampling of 23 buildings: (**a**) Normal probability and (**b**) detrended Normal Q–Q Plot for concrete.

The normal probability and detrended normal Q-Q plots for 25 and 23 structures are shown in Figs. 4 and 5, respectively. The normal probability plots showed a random distribution along the diagonal line, with data points evenly dispersed relative to the horizontal axis.

**Fig. 6:**
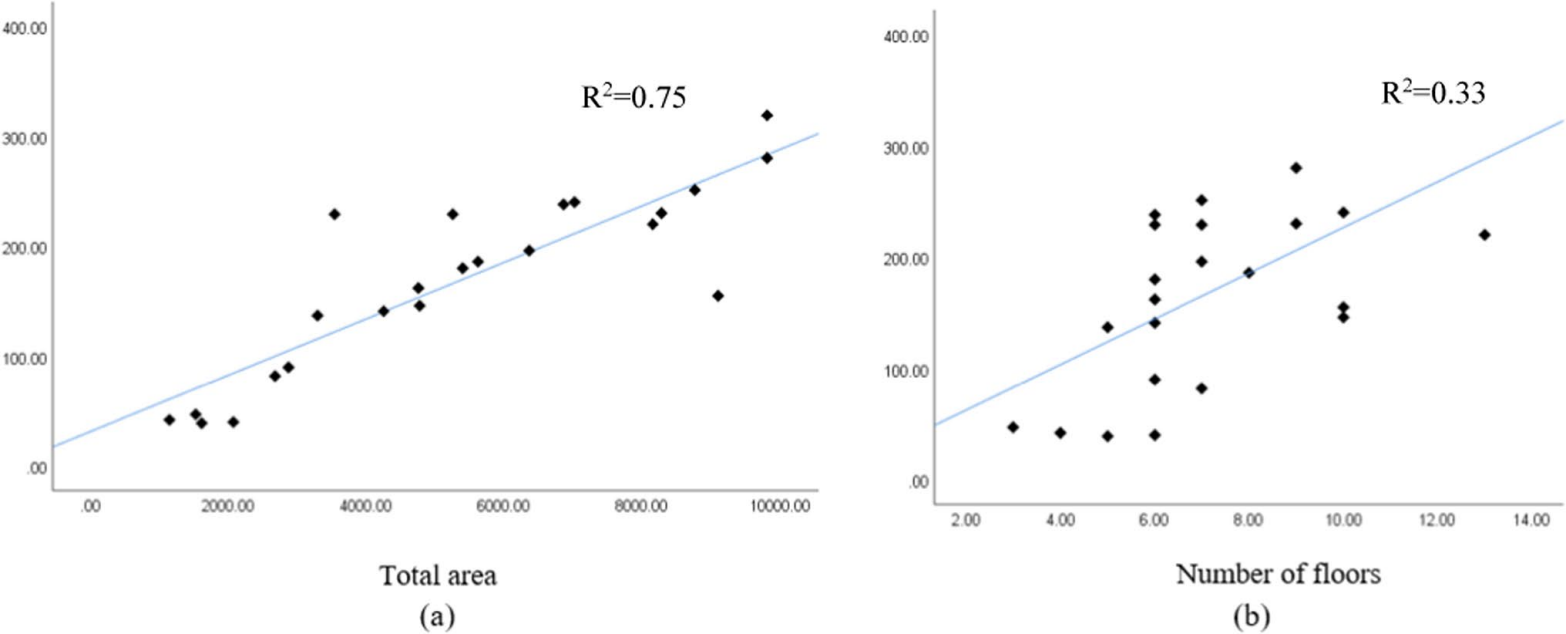
(**a**) Total area and (**b**) number of floors.

The independent variables used for the concrete waste prediction model are total area, number of floors, design consistency, site organization, the experience of workers, and waste reuse. To illustrate cause-and-effect correlations, various independent variables’ dispersion graphs were plotted against the amount of concrete waste. Figure 6 shows the correlation between the variables’ total area, number of floors, and amount of concrete waste (ton).

**Fig. 7:**
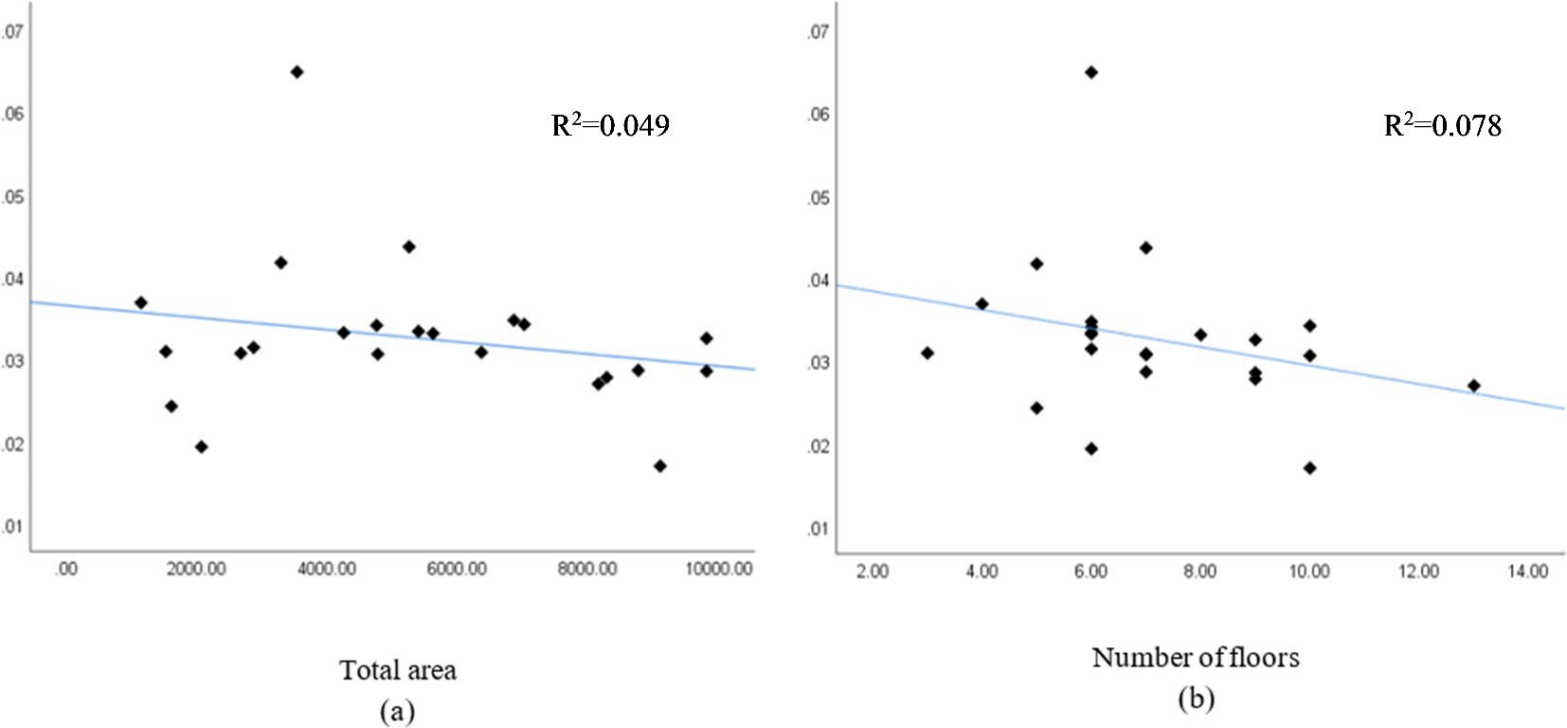
Concrete waste generation and (**a**) total area and (**b**) the number of floors.

Figure 6 shows a positive correlation between the total area, the number of floors, and the amount of concrete waste, indicating that larger total areas and more floors result in more significant amounts of concrete waste. Figure 7 shows dispersion graphs between the total area, the number of floors, and the concrete waste generation ratio, representing the total waste produced ratio to the total area (ton/m^2^). It was noted that the quantity of waste decreased with an increase in the total area and the number of floors. This suggests that employing typical floor plans reduces waste generation due to improved efficiency in construction operations for each additional floor, as indicated in the charts.

Another crucial method for examining relationships among variables is the correlation analysis. It assesses the degree and direction of positive or negative correlation and identifies multicollinearity, where two variables may explain the same phenomenon, potentially affecting the regression procedure’s standard deviation. Table 7 shows Pearson correlation values for the variables.

**Table 7:** Independent variables for concrete waste prediction model – Pearson correlation.

	“Total area”	“Number of floors”	“Design consistency”	“Site organization”	“Experience of workers”	“Waste reuse”
“Total area”	1	0.737**	− 0.524*	− 0.137	− 0.347	− 0.578
“Number of floors”	0.737**	1	− 0.402	− 0.171	− 0.211	− 0.264
“Design consistency”	− 0.524*	− 0.402	1	0.084	− 0.169	0.238
“Site organization”	− 0.137	− 0.171	0.084	1	0.360	0.268
“Experience of workers”	− 0.347	− 0.211	− 0.169	0.360	1	0.453*
“Waste reuse”	− 0.578	− 0.264	0.238	0.268	0.453*	1

*Correlation is significant at the 0.05 level (2-tailed); **Correlation is significant at the 0.01 level (2-tailed).

After conducting correlation analysis, strong multicollinearity between “total area” and “number of floors” (r = 0.737) was identified, indicating redundancy if both variables were included in the regression model. Based on these findings, “total area” was selected for inclusion in the model due to its stronger correlation with “concrete waste” (r = 0.867), suggesting it has a more significant impact on variations in “concrete waste” compared to “number of floors” (r = 0.575). Also, excluding “number of floors” did not significantly impact the model’s precision. This decision helps mitigate issues associated with multicollinearity and ensures a more precise interpretation of predictor effects in the regression analysis.

Effective organization and coordination of site activities are crucial factors influencing concrete waste outcomes^51^. While the statistical significance of “site organization” in the regression model was marginal (*p* = 0.083), its inclusion remains justified due to its theoretical importance and potential impact on waste management strategies.

#### Regression analysis

After confirming the dependent variable’s normality using regression analysis, numerous combinations of independent variables were examined to reach the optimal regression model for a significance level of α = 0.05. All variables passed the F test, and the best regression model (Eq. 1) derived from the sample data had an adjusted R^2^ value of 0.877. The variables “total area” and “waste reuse” were significant at α = 0.05. Equation (1) presents the regression model.

1 $$\begin{aligned} {\text{Concrete waste amount}} & = {128}.{622} + \left( {0.018 \times \hbox{``}{\text{total area}}\hbox{''}} \right) \\ & \quad + \left( { - {11}.{364} \times \hbox{``}{\text{site organization}}\hbox{''}} \right) \\ & \quad + \left( { - {63}.{111} \times \hbox{``}{\text{waste reuse}}\hbox{''}} \right) + \upvarepsilon \\ \end{aligned}$$

**Fig. 8:**
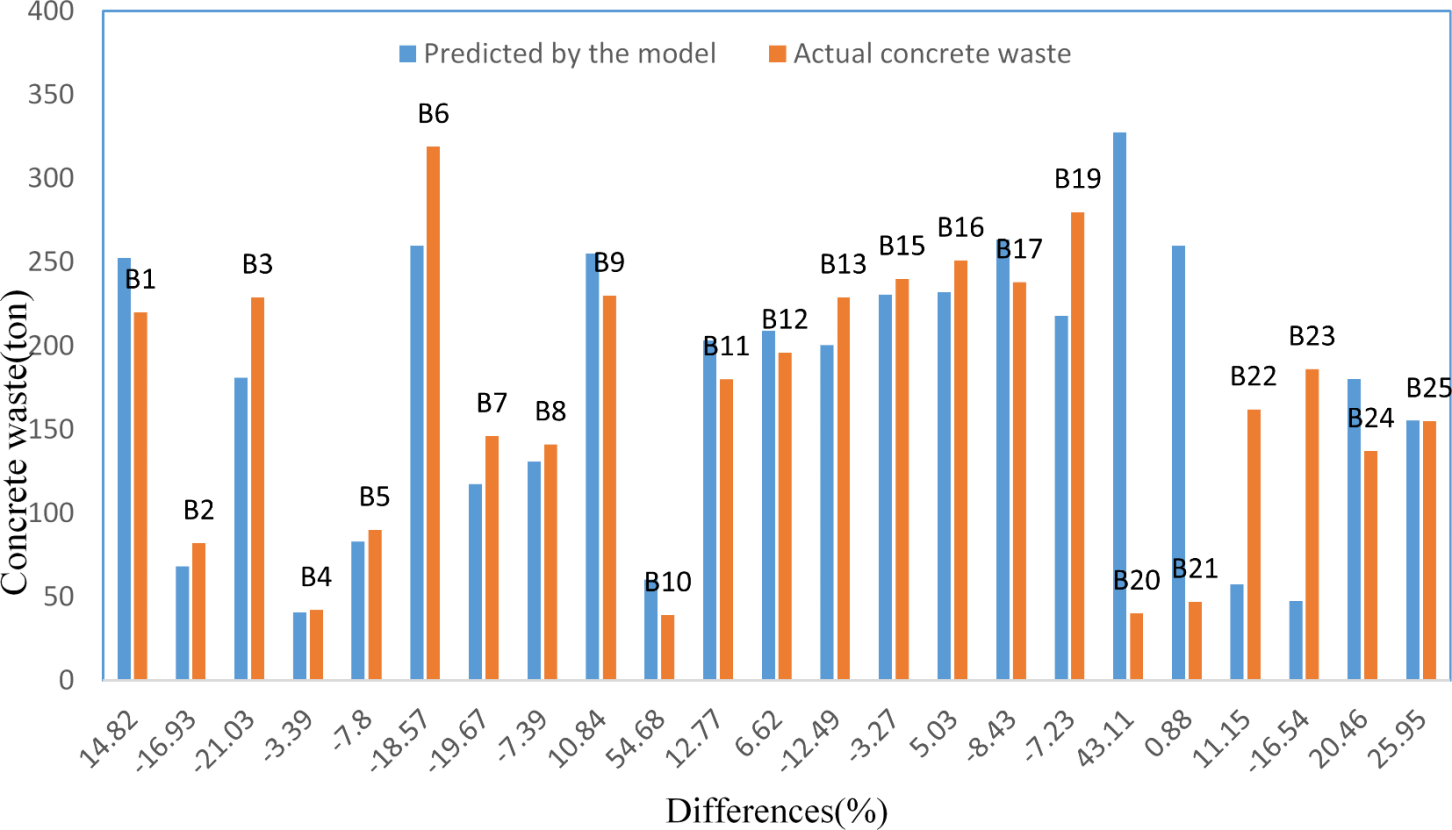
Comparison of predicted vs. actual concrete waste.

Figure 8 compares the concrete waste amounts gathered from sites for 23 buildings with those predicted by the regression model suggested in this study. For 21 buildings (91.3% of the sample), the difference between the actual and predicted amounts of waste was less than 26%. The difference was below 5% in three buildings, indicating a high level of model accuracy. However, the most significant difference was found in building B10 (+ 54.86%), possibly due to project-related issues, such as inadequate shuttering of reinforced concrete columns. On the other hand, the lowest difference was in building B21 (-0.88%).

### Brick prediction model

#### Statistical analysis

**Fig. 9:**
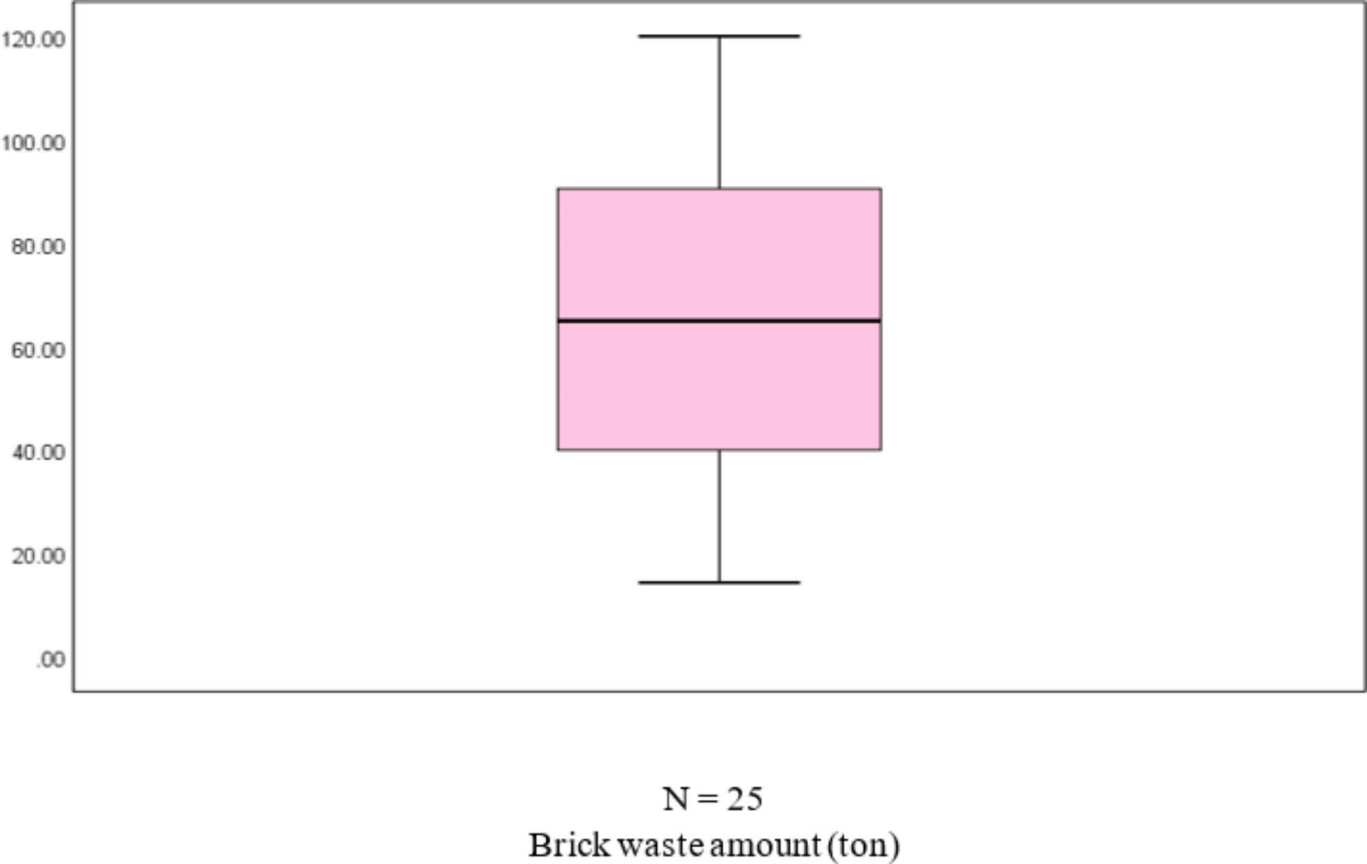
Box plot of the dependent variable.

**Fig. 10:**
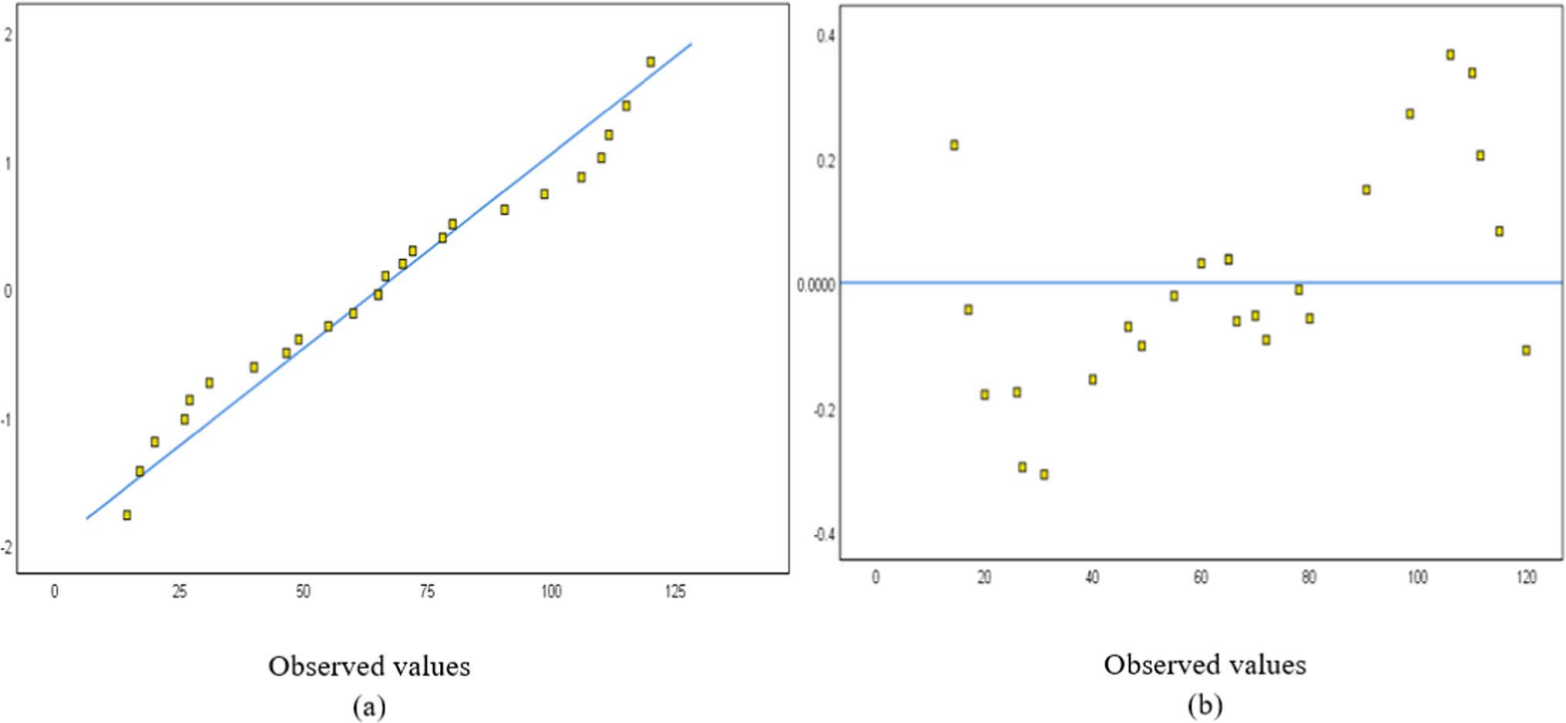
(**a**) Normal probability and (**b**) detrended Normal Q–Q Plot of brick.

As shown in Table 8, the *p* value is 0.255, so the normality hypothesis of the dependent variable failed to be rejected. There are no outliers in the sample, as indicated by the box plot in Fig. 9. The variable’s normal distribution is shown in Fig. 10. Figure 10a shows that the plots are close and have a random distribution along the line. Figure 10b shows that the dispersion of the data points is randomly distributed around the horizontal line, indicating the normality of the sample.

**Table 8:** Normality tests of the dependent variable (brick waste amount).

	Kolmogorov–Smirnov			Shapiro–Wilk		
	Statistic	df	Sig.	Statistic	df	Sig.
Amount of brick waste	0.092	25	0.200	0.950	25	0.225

**Fig. 11:**
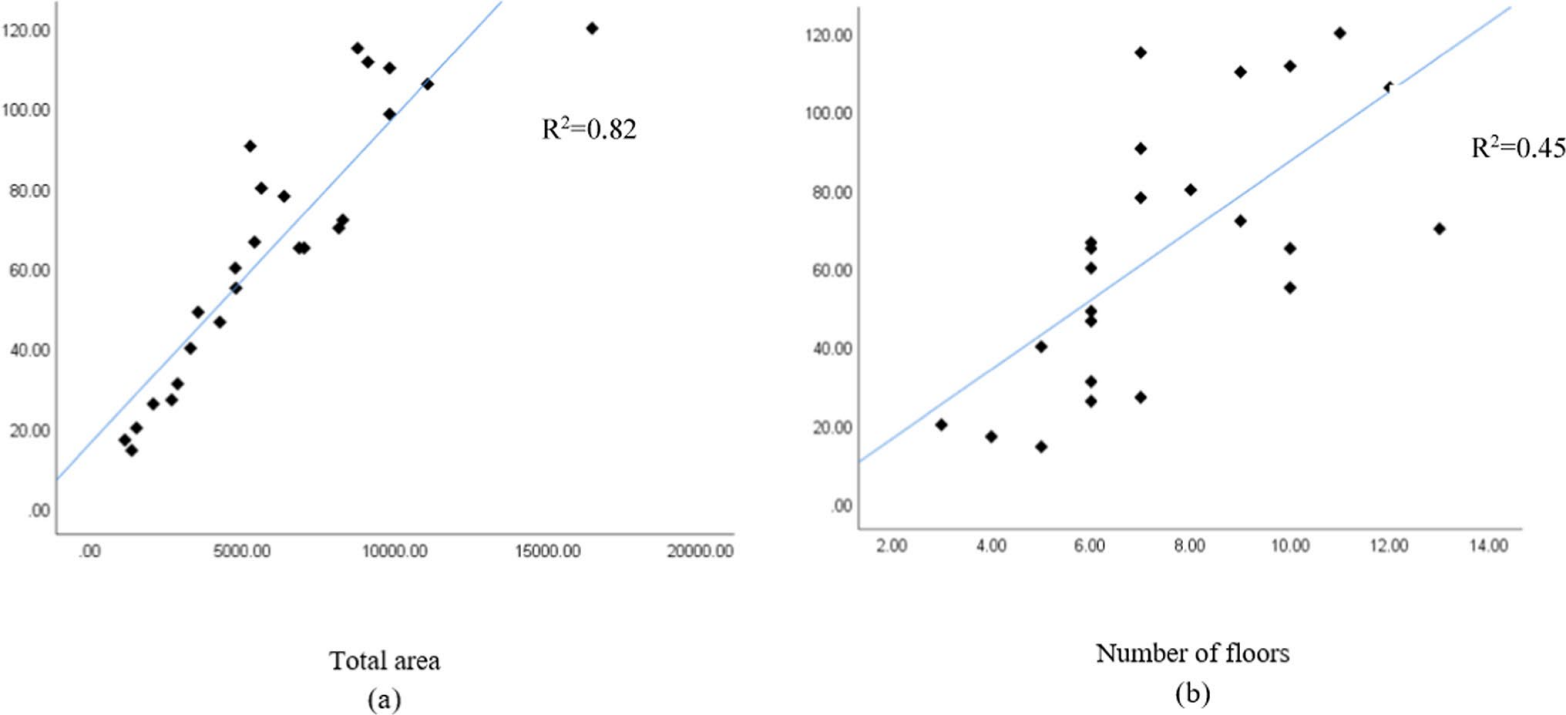
(**a**) Total area and (**b**) number of floors.

**Fig. 12:**
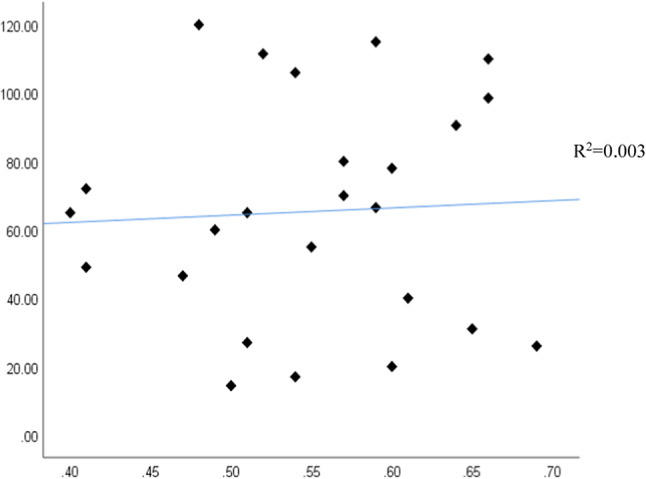
Wall density.

The independent variables used for the brick waste prediction model are total area, number of floors, wall density, design consistency, site organization, the experience of workers, storage of material, and waste reuse. Figures 11 and 12 show the correlation between total area, number of floors, wall density, and brick waste (ton). Figures 11 and 12 show that the greater the total area, number of floors, and wall density, the greater the amount of brick waste.

Correlation analysis was then used to check the relationships between the variables. As shown in Table 9, a higher positive correlation of 0.770 was found between “total area” and “number of floors”. In contrast, a negative correlation of 0.469 was found between “number of floors” and “storage of material”, so the “number of floors” variable was excluded to avoid multicollinearity and ensure a more precise interpretation of predictor effects in the regression analysis.

**Table 9:** Independent variables for brick waste prediction model – Pearson correlation.

	“Total area”	“Number of floors”	“Wall density”	“Design consistency”	“Site organization”	“Experience of workers”	“Storage of material”	“Waste reuse”
“Total area”	1	0.770**	− 0.106	− 0.331	− 0.045	− 0.147	− 0.444	− 0.239
“Number of floors”	0.770**	1	− 0.149	− 0.371	− 0.108	− 0.112	− 0.469*	− 0.108
“Wall density”	− 0.106	− 0.149	1	− 0.137	0.421*	− 0.419*	0.187	0.065
“Design consistency”	− 0.331	− 0.371	− 0.137	1	0.077	0.437*	0.026	0.026
“Site organization”	− .045	− 0.108	0.421*	0.077	1	0.157	− .091	0.245
“Experience of workers”	− 0.147	− 0.112	− 0.419*	0.437*	0.157	1	− 0.418*	0.247
“Storage of material”	− 0.444*	− 0.469*	0.187	0.026	− 0.091	− 0.418*	1	0.188
“Waste reuse”	− 0.239	− 0.108	0.065	0.026	0.245	0.247	0.188	1

*Correlation is significant at the 0.05 level (2-tailed); **Correlation is significant at the 0.01 level (2-tailed).

“Waste reuse” did not have significant correlations with other variables, indicating it only accounts for a single factor. Consequently, this variable was excluded from the regression analysis to ensure the model focuses on variables that demonstrate more robust associations with brick waste. The “design consistency” and “storage of material” variables were not significant in this model, but they showed a significant correlation with other variables, suggesting potential overlap in their impact on brick waste generation.

#### Regression analysis

Multiple combinations of independent variables were tested to find the optimal regression model. All variables passed the F test, and the optimal regression model (Eq. 2) derived from the sample data had an adjusted R^2^ value of 0.893. The variables “total area” and “experience of workers” were significant for α = 0.05. The best regression model is presented in Eq. (2).

2 $$\begin{aligned} {\text{Brick waste amount}} & = {63}.{339} + \left( {0.00{7} \times \hbox{``}{\text{total area}}\hbox{''}} \right) \\ & \quad + \, \left( { - {7}.0{55} \times \hbox{``}{\text{design consistency}}\hbox{''}} \right) \\ & \quad + \, \left( { - {11}.{5}0{8} \times \hbox{``}{\text{experience of workers}}\hbox{''}} \right) \\ & \quad + \, \left( { - {9}.{596} \times \hbox{``}{\text{storage of material}}\hbox{''}} \right) + \upvarepsilon \\ \end{aligned}$$

**Fig. 13:**
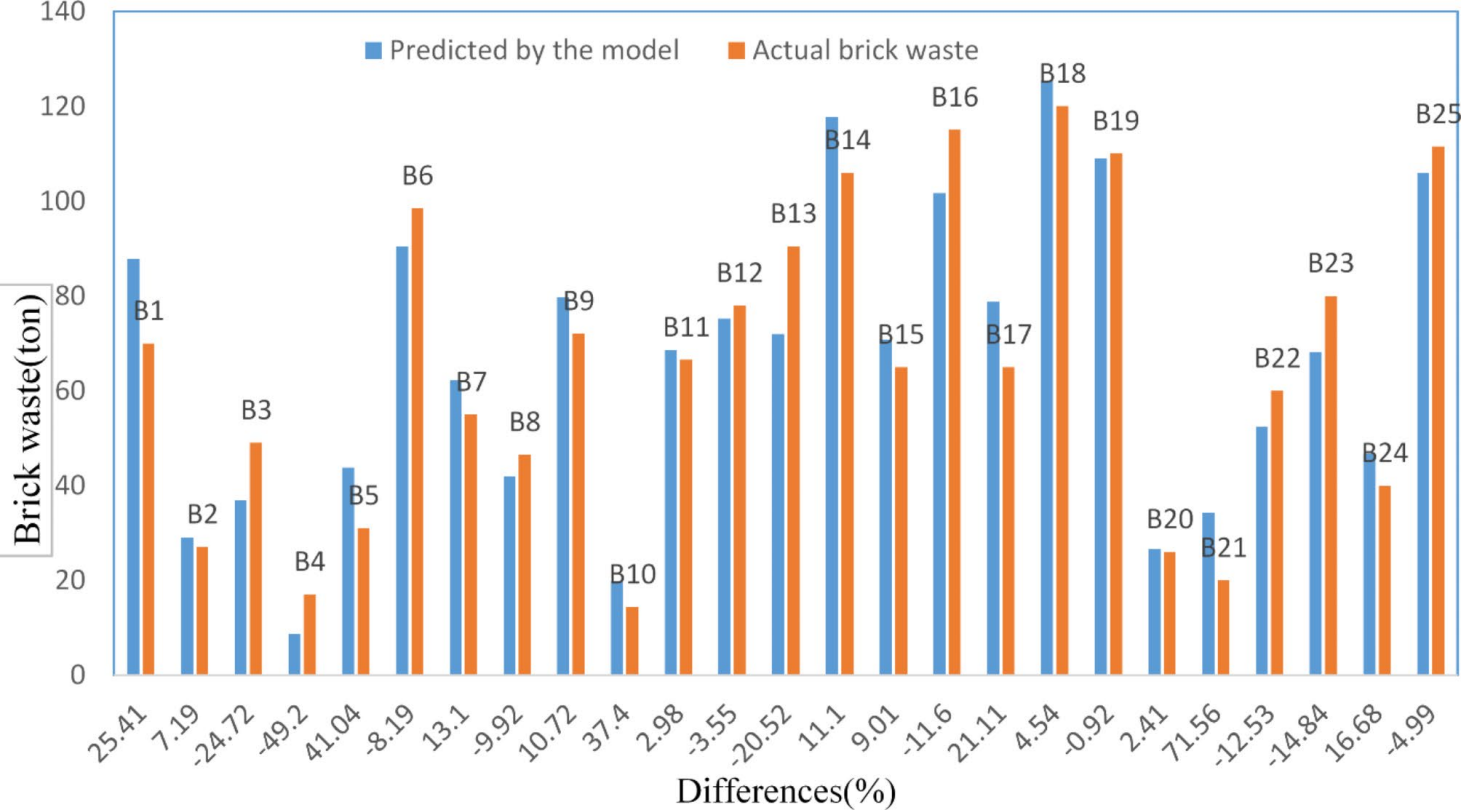
Comparison of predicted vs. actual brick waste.

Figure 13 compares the amount of brick waste from the 25 buildings gathered from sites with the amount predicted by the regression model suggested in this study. For 20 buildings (80% of the sample), the difference between the actual and predicted amounts of waste was less than 25%. The difference was below 5% in five buildings. The most significant difference was found in building B21 (+ 71.56%), while the lowest was in building B19 (-0.92%).

### Steel prediction model

#### Statistical analysis

**Fig. 14:**
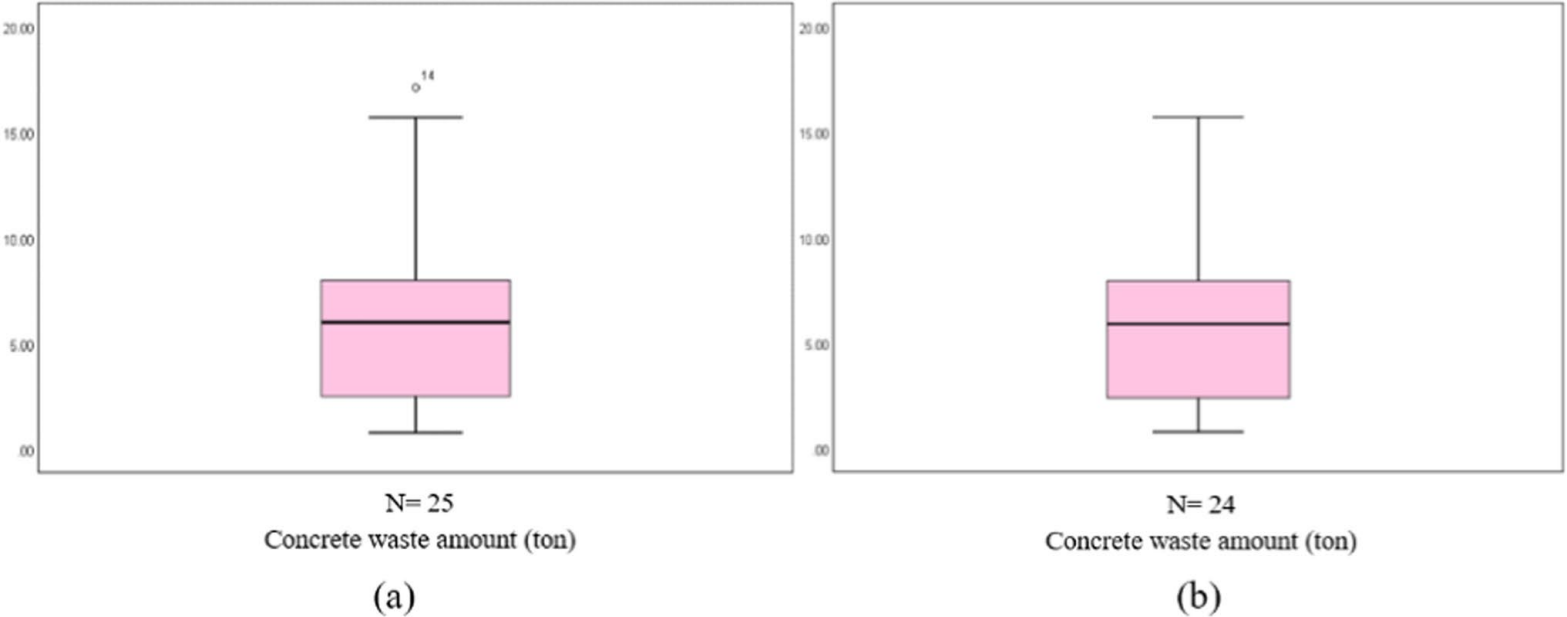
(**a**) Box plot of the 25 buildings, and (**b**) box plot of the 24 buildings.

The normality hypothesis for the dependent variable was rejected, as the p-value was 0.026. The box plot in Fig. 14a also indicates an outlier in the sample (B14). After identifying and excluding the outlier from the dataset, the normality tests of 24 building samples were conducted, with a *p* value of 0.06, so the normality hypothesis of the dependent variable was not rejected. There are no outliers in the 24 building samples, as indicated by the box plot in Fig. 14b. Table 10 compares the two sample sets’ normality tests before and after removing outliers.

**Table 10:** The dependent variable’s normality tests (25 and 24 building samples) for steel.

	Kolmogorov–Smirnov			Shapiro–Wilk		
	Statistic	df	Sig.	Statistic	df	Sig.
25 buildings						
Steel waste amount	0.126	25	0.120	0.907	25	0.026
24 buildings						
Steel waste amount	0.132	24	0.200	0.921	24	0.060

**Fig. 15:**
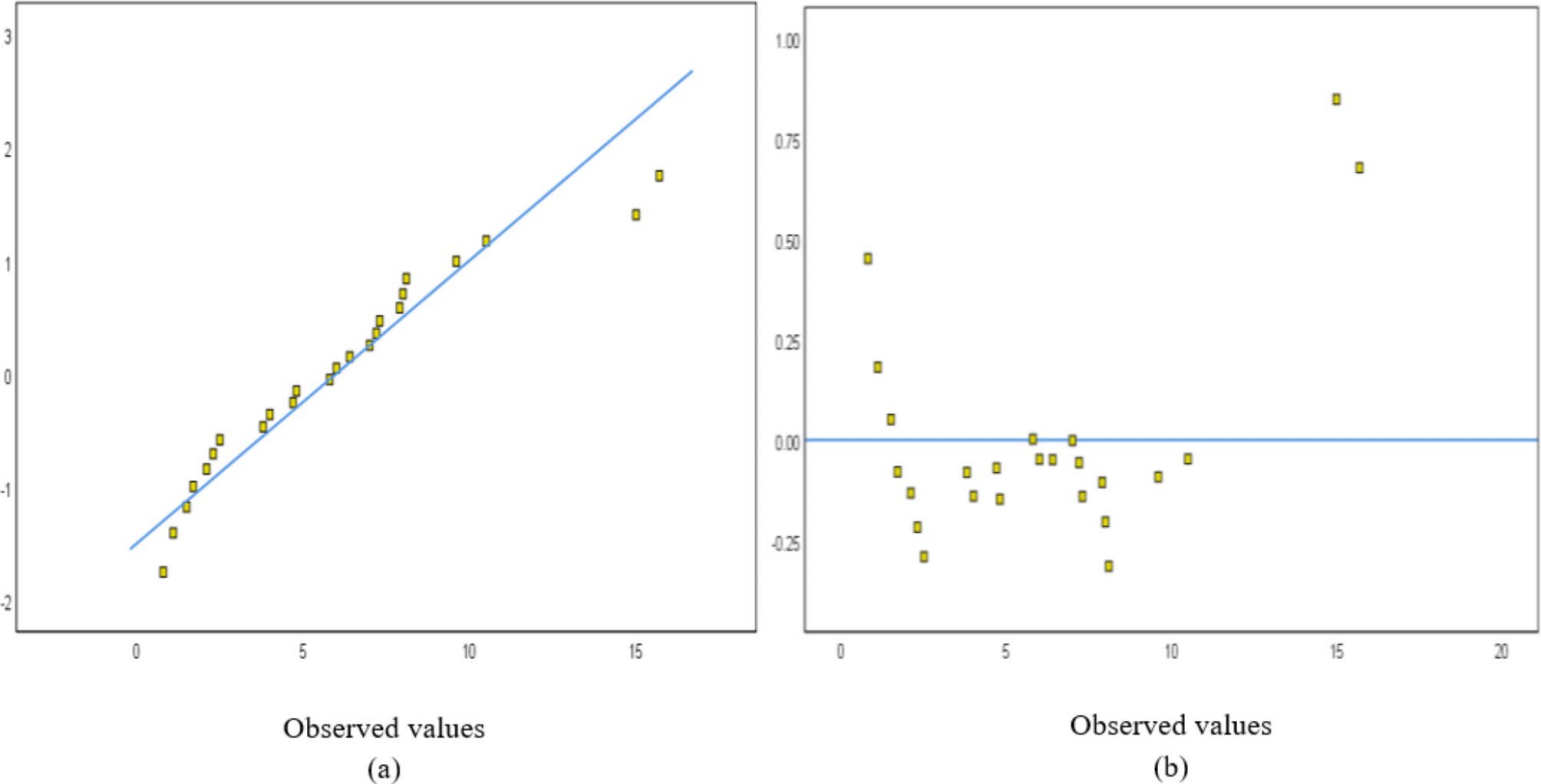
A sampling of 25 buildings: (**a**) Normal probability and (**b**) detrended Normal Q–Q Plot for steel.

**Fig. 16:**
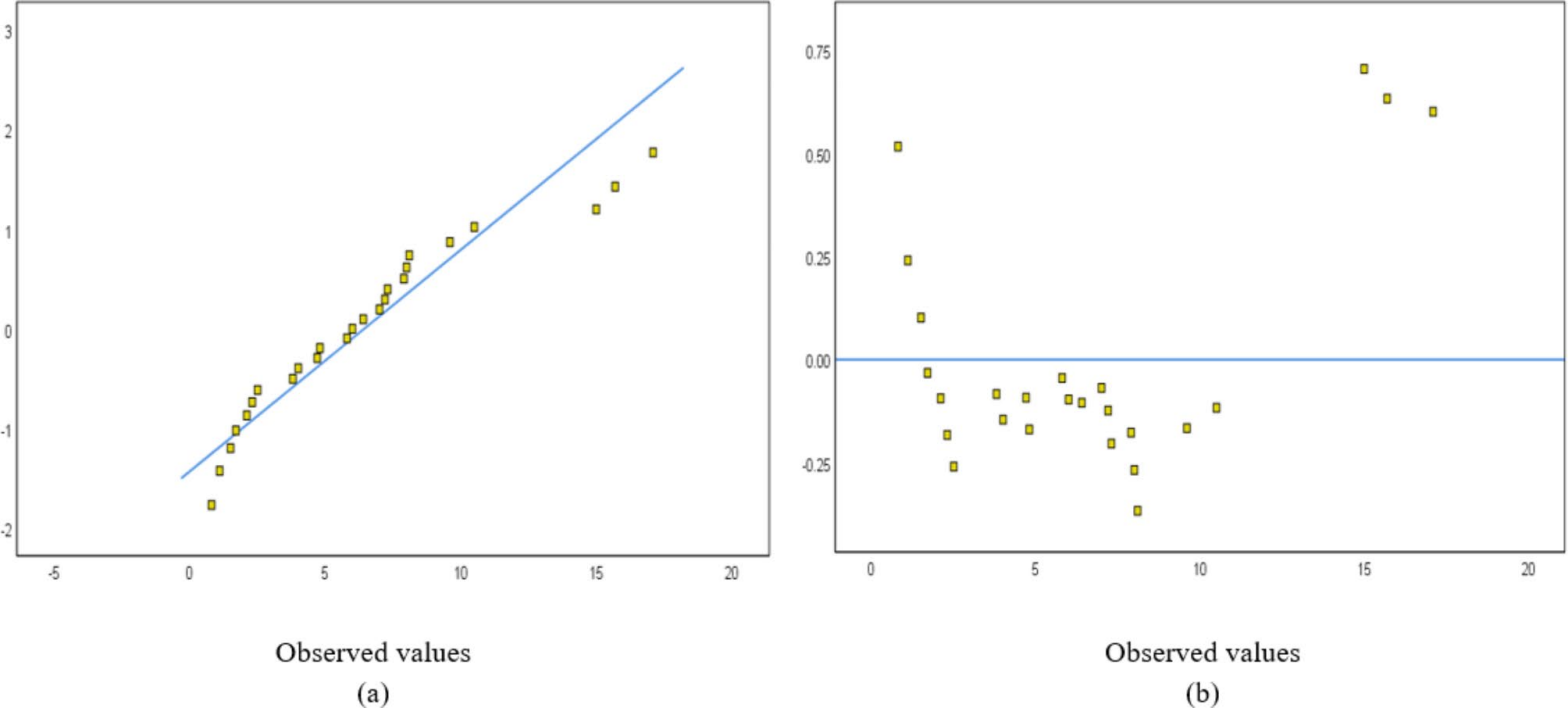
A sampling of 24 buildings: (**a**) Normal probability and (**b**) detrended Normal Q–Q Plot for steel.

**Fig. 17:**
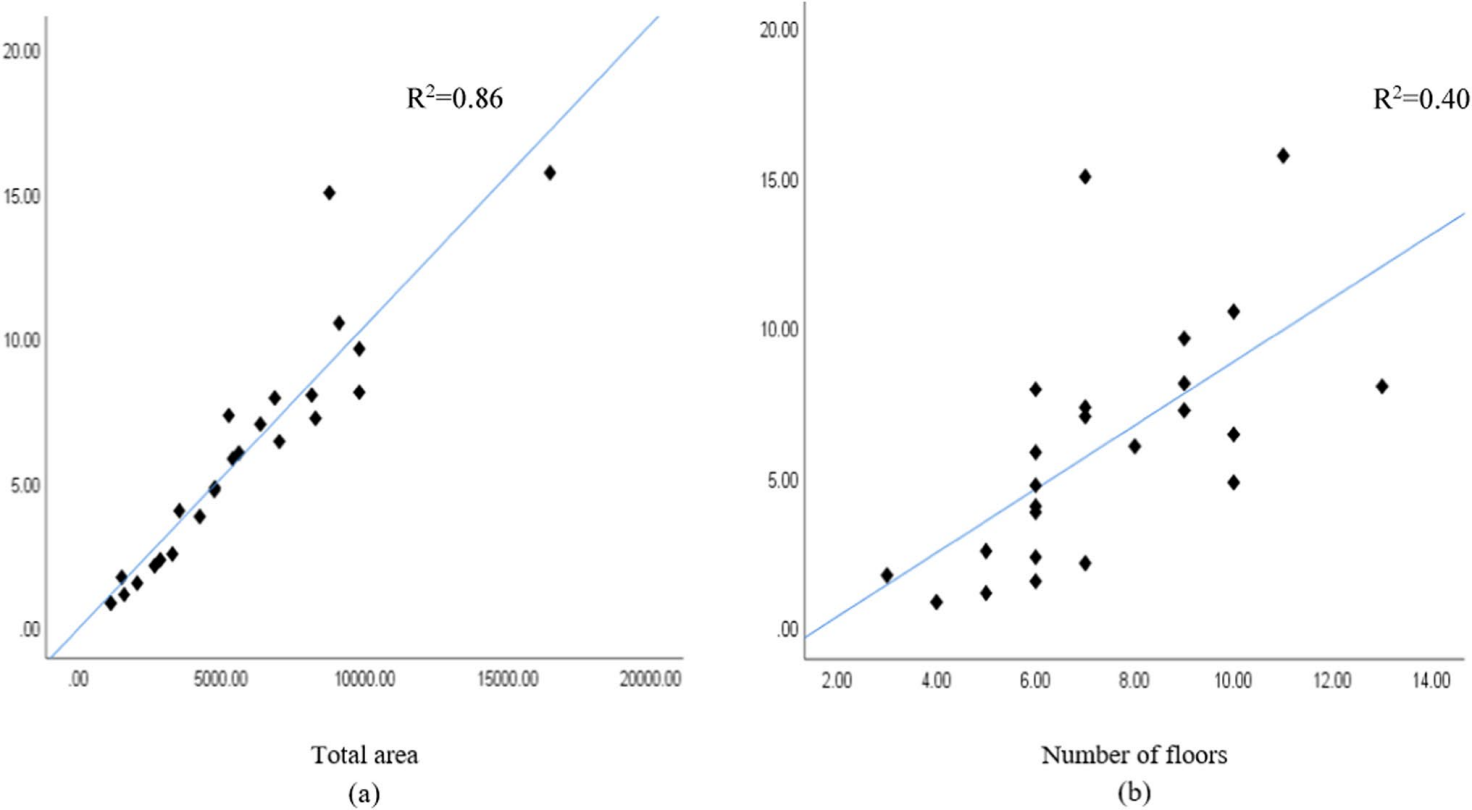
(**a**) Total area and (**b**) number of floors.

The normal probability and detrended normal Q–Q plots for 25 and 24 structures are shown in Figs. 15 and 16, respectively. The normal probability plots also showed a random distribution along the line, with data points evenly distributed around the horizontal line. The independent variables used for the steel waste prediction model are total area, number of floors, design consistency, site organization, the experience of workers, storage of material, and waste reuse. Figure 17 shows the correlation between the variables’ total area, number of floors, and amount of steel waste (ton).

Figure 17 shows a positive correlation between the total area and number of floors with the amount of steel waste, indicating that larger total areas and more floors result in more significant amounts of steel waste.

As shown in Table 11, “site organization” was found to have the lowest impact on steel waste generation. This variable did not have significant correlations with other variables, indicating it only accounts for a single factor, so this variable was omitted from the regression analysis to ensure the model focuses on variables that demonstrate more robust associations with steel waste. In this model, the “number of floors” and “waste reuse” variables were not significant, but they showed a significant correlation with other variables, suggesting potential overlap in their impact on steel waste generation.

**Table 11:** Independent variables for steel waste prediction model – Pearson correlation.

	“Total area”	“Number of floors”	“Design consistency”	“Site organization”	“Experience of workers”	“Storage of material”	“Waste reuse”
“Total area”	1	0.770**	− 0.330	− 0.045	− 0.691**	− 0.448*	− 0.619**
“Number of floors”	0.770**	1	− 0.371	− 0.108	− 0.542**	− 0.469*	− 0.360
“Design consistency”	− 0.330	− 0.371	1	0.077	0.485*	0.026	0.099
“Site organization”	− .045	− 0.108	0.077	1	0.108	− .091	0.102
“Experience of workers”	− 0.691**	− 0.542**	0.485*	0.108	1	0.485*	0.650**
“Storage of material”	− 0.448*	− 0.469*	0.026	− 0.091	0.485*	1	0.428*
“Waste reuse”	− 0.619**	− 0.360	0.099	0.102	0.650**	0.428*	1

*Correlation is significant at the 0.05 level (2-tailed); **Correlation is significant at the 0.01 level (2-tailed).

#### Regression analysis

Many combinations of the independent variables were analyzed to select the best regression model. All variables passed the F test, and the optimal regression model (Eq. 3) derived from the sample data had an adjusted R^2^ value of 0.889. The variables “total area” and “design consistency” were significant for α = 0.05. The best regression model is presented in Eq. (3).

3 $$\begin{aligned} {\text{Steel waste amount}} & = {3}.{787} + \left( {0.00{1} \times \hbox{``}{\text{total area}}\hbox{''}} \right) \\ & \quad + \left( { - 0.{265} \times \hbox{``}{\text{number of floors}}\hbox{''}} \right) \\ & \quad + \left( { - {1}.{578} \times \hbox{``}{\text{design consistency}}\hbox{''}} \right) \\ & \quad + \left( { - {1}.{21} \times \hbox{``}{\text{waste reuse}}\hbox{''}} \right) + \upvarepsilon \\ \end{aligned}$$

**Fig. 18:**
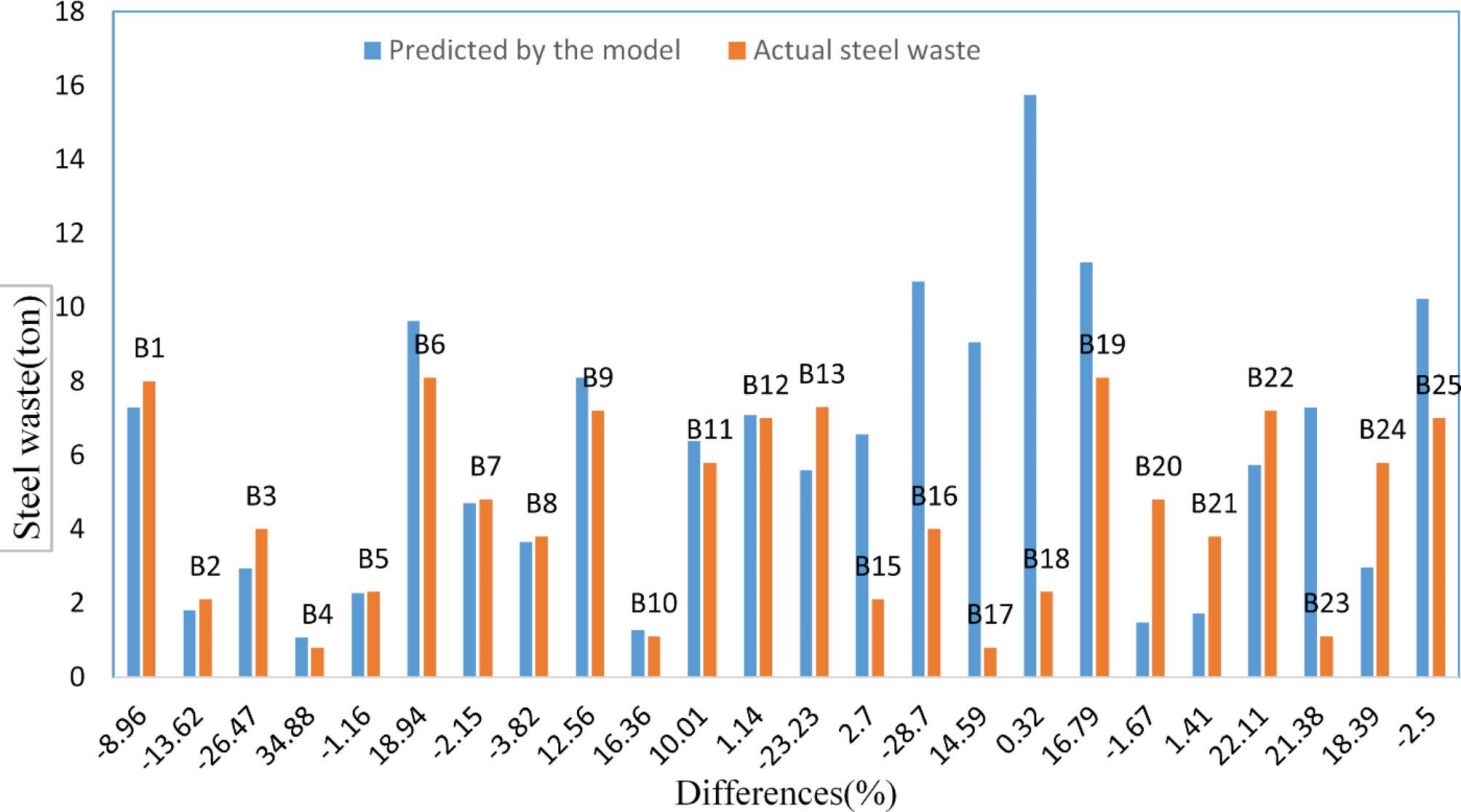
Comparison of predicted vs. actual steel waste.

Figure 18 compares the amount of steel waste gathered from sites in 24 buildings with the amount predicted by the regression model suggested in this study. For 21 buildings (87.5% of the sample), the difference between the actual and predicted amounts of waste was less than 25%. The difference was below 5% in eight buildings. The most significant difference was found in building B4 (+ 34.88%), while the lowest was in building B18 (-0.32%).

## Discussion

In this study, statistical multiple regression models were developed to predict the quantity of concrete, brick, and steel waste generated at construction sites. The analysis in this study focused on variables related to design and site management attributes. These variables are widely recognized in the literature as significant contributors to CW. The variable “total area” emerged as a significant predictor in all three models developed for predicting concrete, brick, and steel waste generation. Its consistent significance shows its critical role in influencing waste outcomes in construction projects. This finding aligns with prior research emphasizing “total area” as a critical determinant of waste generation^6,18,38^. Although the number of floors was not statistically significant in the steel model (*p* value = 0.155), its inclusion improved the model’s results. Furthermore, statistical linear regression indicates that the variables act together. For instance, in the concrete prediction model, the adjusted R^2^ decreased when the variable “experience of workers” was removed, even though this variable was not statistically significant at the adopted significance level.

Among all the tested variables, those that were seen to affect concrete waste generation included “total area”, “site organization”, and “waste reuse”; and on brick waste generation, “total area”, “design consistency”, “experience of workers” and “storage of material”; and on steel waste generation, “total area”, “number of floors”, “design consistency”, and “waste reuse”. “Site organization” was not found to be a significant predictor in the brick and steel waste models since its p-value was higher than α = 0.05. This lack of statistical significance shows that within the dataset used in this study, “site organization” did not contribute substantially to explaining the variation in brick and steel waste generation.

The models achieved high adjusted R^2^ of 0.877, 0.893, and 0.889 for concrete, brick, and steel waste prediction, respectively. These R^2^ values indicate that the models explain approximately 88% to 89% of the variance in waste generation in residential buildings in the sample. These results statistically demonstrate the impact of these variables on waste generation in residential buildings. This statement was validated through the regression model used to predict the amount of waste generated in the sample buildings compared to the amount gathered from sites (see Figs. 8, 13, and 18).

When these results are compared with studies mentioned in the literature^6,11,18,29,38,52^, many similarities regarding the model structure and built-in features can be identified. The R^2^ values in these studies fall within the range of 0.694 to 0.999, closely matching the results reported in Figs. 8, 13, and 18, and offer more accurate predictions than the linear prediction model developed by Parisi Kern et al.^11^, which had an R^2^ value of 0.694 Similar interpretations can be made when searching in Hassan et al.^38^, where they implemented the regression model (R^2^ = 0.78) for the brick waste generation prediction. The results indicate that the statistical regression models effectively estimated the amount of concrete, brick, and steel waste generated in concluded projects. For future projects, decision-makers can use these models proactively. For example, during the project planning phase, the models can forecast waste generation based on key project characteristics (e.g., total area, number of floors, and site organization). By identifying expected waste volume in concrete, brick, and steel areas, project managers can allocate resources more efficiently by allocating, such as adequate storage areas, segregation zones, and transportation logistics. These models enable the simulation of variables, providing insights into how design and site management decisions can influence either a decrease or increase in waste generation.

While the developed models provide valuable insights into CW prediction, it is crucial to assess their limitations to improve their applicability and reliability. The linearity assumption built into linear regression models is one major issue. However, waste generation processes associated with complex relationships among multiple variables, including site organization, design consistency, and the experience of workers, cannot always be represented by a linear relationship. Another limitation is that it only relies on the quality and range of input data. The models are developed based on the gathered data obtained from 25 construction projects in Egypt, which may limit their generalizability in other areas with different construction methodologies, policies on construction, and usage of materials. Future research could explore integrating advanced technologies such as artificial intelligence (AI) and machine learning. AI-based approaches, such as adaptive neuro-fuzzy systems, support vector machines, or random forests, can capture non-linear relationships between variables in the data, potentially enhancing prediction precision.

## Conclusion

CW constitutes a substantial share of the total waste generated by the community, and finding methods to reduce CW generation remains a significant objective in the construction industry. This study enhances the existing knowledge about waste generation at construction sites by applying statistical modeling. A method for measuring waste has been implemented, and data were gathered from several construction building sites. To validate these models, the amount of waste collected from sites was compared with the predictions made by the regression models. The analysis revealed discrepancies in the predictions, ranging from + 54.86% (largest) to − 0.88% (smallest) for concrete, + 71.56% (largest) to − 0.92% (smallest) for brick, and + 34.88% (largest) to − 0.32% (smallest) for steel. Notably, the differences were less than 5% in three buildings for the concrete model, five for the brick model, and eight for the steel model. These variations highlight the models’ strengths and limitations in accurately forecasting waste quantities.

The regression models proposed demonstrated acceptable statistical performance, suggesting they could be satisfactory for estimating waste generation to inform management plans. The models suggest a comprehensive relationship between waste generation and buildings’ characteristics. This study examined sources of waste generation identified in the literature, focusing on design and site management attributes across 25 residential buildings using statistical regression models.

Future studies should expand data collection to include a broader range of building types (e.g., commercial and industrial) and sizes to improve the models. Additionally, considering factors such as construction methods, quality management on site, and contractor practices can capture a more comprehensive picture of waste generation. Updating the models with new data, such as data from different building types (e.g., commercial, industrial, mixed-use, and infrastructure), to reflect changes in construction practices and material usage over time might further refine the models.

In summary, the regression analysis-based models enhance the understanding of waste generation by highlighting the most significant features and their respective influences. Consequently, these models can forecast the waste generation in projects with similar features. The predictive models developed in this study offer several key benefits, such as improved waste management, cost savings, enhanced sustainability, and informed decision-making. By predicting waste before construction begins, they assist builders in enhancing onsite waste management. This approach can reduce waste in new projects, supporting more sustainable construction practices. In addition to their practical significance, these models enrich the overall concept of sustainable building construction. Therefore, they are valuable for enabling decision-makers to implement relevant interventions toward realizing circular economy strategies like material reuse and recycling based on critical waste determinants that cause an increase in waste. Furthermore, using the above-mentioned predictive models in the project’s planning can help improve compliance with the existing sustainability laws, minimize environmental negative impacts through controlling carbon emissions, and promote eco-friendly construction techniques.

Egyptian construction waste management policies may significantly affect the construction industry. They consist of enhancing waste and recycling disposal laws, promoting financial incentives regarding sustainable construction, and the requirement for environmental impact studies on massive construction projects. Several recommendations are put forward to enhance waste tracking and operation efficiency, including increasing the application of the latest technologies like AI and prefabrication. These measures would promote sustainability, save materials, and improve industry productivity.

## Data Availability

Data will be made available on request from the corresponding authors.
